# Unexpected Outcomes of CDK4/6 Inhibition

**DOI:** 10.18632/oncotarget.912

**Published:** 2013-02-27

**Authors:** Yoon Jong Choi, Piotr Sicinski

**Affiliations:** Department of Cancer Biology, Dana-Farber Cancer Institute and Department of Genetics, Harvard Medical School, Boston, Massachusetts, USA; Department of Cancer Biology, Dana-Farber Cancer Institute and Department of Genetics, Harvard Medical School, Boston, Massachusetts, USA

The response of cells to extracellular mitogenic signals and commitment to enter G1 phase, are regulated by the D-type cyclins (D1, D2 and D3). Once induced they heterodimerize with and activate either cyclin dependent kinase 4 or 6 (CDK4 or CDK6). Cyclin D-CDK4 and D-CDK6 kinases phosphorylate and inactivate the retinoblastoma family of proteins, leading to release and derepression of E2F transcription factors. E2Fs, in turn, activate a transcriptional program required for G1-S transition. Cyclin D-associated kinase also affects other pathways by phosphorylating SMADs and FOXM1 transcriptional regulators. In addition, cyclin D-CDK4/6 complexes play an important non-catalytic function by sequestering cell cycle inhibitors p21^Cip1^ and p27^Kip1^ from CDK2 [1].

Dysregulated expression of D-cyclins and resulting CDK4/6 hyperactivation are thought to represent a driving force in tumorigenesis. Indeed, cyclin D1 and CDK4 are among the most commonly amplified genes across all human cancer types [2]. Cyclin D1 gene is amplified in up to 20% of invasive breast cancers, while the protein is overexpressed in over 50% of mammary carcinomas [1]. Cyclin D3 is highly expressed in several hematopoietic malignancies, such as T-cell acute lymphoblastic leukemias (T-ALL). These observations suggest that cyclin D-CDK4 and D-CDK6 kinases might represent attractive therapeutic targets in cancer treatment. Recent analyses using mouse cancer models revealed unexpected outcomes of cyclin D-CDK kinase inhibition *in vivo*, in tumor bearing mice.

Cyclin D germline knockout animals provided some of the first evidence that the D-type cyclins are required for tumor initiation. Thus, several groups have shown that mice lacking cyclin D1, or lacking CDK4, or expressing kinase-inactive cyclin D1-CDK complexes are either completely resistant, or show significantly reduced susceptibility to HER2-driven breast cancers, depending on the genetic background used (see references in [3]). Likewise, cyclin D3-deficient mice, or animals lacking CDK6 are resistant to T-cell acute lymphoblastic leukemias (T-ALL) driven by the NOTCH1 oncogene, or by its downstream effector AKT (see references in [3]).

It could have been argued that tumor resistance observed in cyclin- or CDK-deficient animals was due to the developmental defects caused by germline cyclin/CDK ablation. Indeed, cyclin D1-knockout mice show defects in mammary epithelial lobuloalveolar development [4]. Likewise hematopoietic abnormalities were noted in cyclin D3- or CDK6-null mice [4]. Collectively, these observations raised a possibility that progenitor cells targeted for transformation might have been missing in knockout animals.

To circumvent the developmental effects of germline cyclin ablation, we generated conditional cyclin D-knockout mouse strains. These strains enabled us to ubiquitously turn off cyclin expression after normal development had been completed. We allowed conditional cyclin D1 knockout animals to undergo normal development in the presence of cyclin D1, and then switched off cyclin D1 expression once mammary development had been completed, and after the animals had developed breast tumors. We found that an acute and ubiquitous shutdown of cyclin D1 in animals bearing HER2-driven breast cancers blocked tumor progression, without having any obvious impact on the animals' health. Likewise, we observed that shutdown of cyclin D3 halted progression of NOTCH1-overexpressing T-ALL [3]. Collectively, these observations established that tumors with specific genetic lesions are dependent on particular cyclin proteins. Moreover, this dependence is not caused by developmental defects observed in knockout animals, but it reflects a rate-limiting function of cyclin proteins in cancer cells.

Inhibition of a critical cell cycle protein would be expected to reversibly arrest tumor cell proliferation. Contrary to this expectation, the Barbacid group demonstrated that an acute ablation of CDK4 in a mouse model of K-RAS driven lung adenocarcinomas resulted in cancer cell senescence [5]. We observed that shutdown of cyclin D1 triggered senescence of HER2-driven breast cancers [3]. Unexpectedly, inhibition of cyclin D3 in a mouse model of NOTCH1-overexpressing T-ALL had a different outcome, namely it caused tumor cell apoptosis [3]. Moreover, administration of a highly specific CDK4/6 inhibitor, PD0332991 to tumor bearing mice essentially phenocopied cyclin D ablation, namely it caused senescence of HER2-positive breast cancer cells and it selectively killed NOTCH1-overexpressing T-ALL cells [3, 6].

Activating mutations in the NOTCH1 pathway are observed in over 50% of T-ALL cases [7]. Our analyses of human cancer cell lines revealed that only NOTCH1-positive cells responded to cyclin D-CDK4/6 inhibition by undergoing apoptosis. In contrast, only cytostatic effects of PD0332991 treatment were observed in other hematological malignancies analyzed (NOTCH1-negative T-ALL, B-cell acute lymphoblastic leukemia, multiple myeloma, mantle cell lymphoma) [3]. Hence, these analyses revealed a synthetic-lethal interaction between NOTCH1 activation and cyclin D-CDK4/6 inhibition.

When the mammalian cell cycle machinery was discovered, it was assumed that its components operate in the same way in all cell types. However, evidence accumulated in the past few years indicates that this is not the case. Germline knockout experiments revealed that individual cell cycle proteins are dispensable for proliferation of the overwhelming majority of cell types [4]. Importantly, these proteins are essential for initiation and maintenance of specific cancer types, depending on the genetic lesions they carry. The observed interactions between CDK4/6 inhibition and mutations within well-characterized oncogenes, reported recently by Puyol et al. [5], Choi et al. [3] and Sawai et al. [6], add to a growing list of cases when inhibition of a cell cycle regulator selectively targets cancer cells. For example, in normal non-transformed cells cyclin dependent kinases CDK1 and CDK2 play a role in S and G2/M phase progression by partnering with cyclins E, A and B. Surprisingly, inhibition of CDK1 was shown to trigger cell death of Myc-overexpressing cancer cells [8]. In contrast, inhibition of a related kinase, CDK2, was demonstrated to cause senescence of Myc-driven tumor cells [9]. Inhibition of CDC7 kinase, which together with its regulatory partners DBF4 and DRF1 is responsible for firing origins of DNA replication, was shown to selectively kill p53-deficient cancer cells [10]. These observations underscore a notion that specific genetic lesions render cancer cells dependent on particular cell cycle proteins not only for proliferation, but also for survival or for protection against senescence. While considerable effort should be invested in elucidating the molecular basis of these dependencies, from the clinical standpoint the most important task is to delineate cancer subtypes in which inhibiting particular cell cycle proteins would selectively target cancer cells. With the introduction of highly specific CDK inhibitors, there is growing hope that targeting selective cell cycle components may offer a very powerful way to specifically eliminate cancer cells.
